# Pin1 as a Central Modulator of Wnt/β-Catenin Signaling in Pulmonary Fibrosis: Interplay with EBV-LMP1 and Therapeutic Implications

**DOI:** 10.5812/ijpr-160860

**Published:** 2025-12-08

**Authors:** Xiaodan Jiao, Jin Yingli, Yanchao Liu, Hong Jiang Yan, Pei Wang, Yadong Yuan

**Affiliations:** 1 Department of Respiratory and Critical Care Medicine, The Second Hospital of Hebei Medical University, Shijiazhuang, China; 2 Department of Emergency, The Second Hospital of Hebei Medical University, Shijiazhuang, China; 3 Department of Thoracic Surgery, The Second Hospital of Hebei Medical University, Shijiazhuang, China

**Keywords:** Pulmonary Fibrosis, Wnt/β-Catenin Signaling, Pin1, Lung Fibroblasts, Signal Transduction, Therapeutic Target

## Abstract

**Background:**

Pulmonary fibrosis (PF) is characterized by dysregulated signaling, with the Wnt/β-catenin pathway playing a critical role. Pin1, a peptidyl-prolyl isomerase, is implicated in post-translational modifications and cellular signaling.

**Objectives:**

This study explores the expression, localization, and functional role of Pin1 in regulating Wnt/β-catenin signaling in human lung fibroblasts (MRC-5 cells). These cells, derived from normal lung tissue, are commonly used to model fibrotic processes due to their ability to mimic fibroblast behavior in PF. Importantly, we report the first demonstration of Epstein-Barr virus (EBV) latent membrane protein 1 (LMP1)-mediated Pin1 activation in the context of PF. Notably, we demonstrate that EBV-LMP1 activates Pin1 and amplifies Wnt/β-catenin signaling in fibroblasts.

**Methods:**

We employed a combination of Pin1 overexpression and siRNA-mediated knockdown in MRC-5 cells to assess pathway modulation. Subcellular localization analysis was performed, and pathway output was evaluated by quantifying β-catenin, cyclin D1, and Axin2 via Western blotting. Co-immunoprecipitation (Co-IP) was used to examine the Pin1-β-catenin interaction. To examine viral contributions, LMP1 overexpression was carried out, and pharmacological inhibition of Pin1 was achieved using Juglone and PiB.

**Results:**

Pin1 expression was significantly higher in MRC-5 cells compared to alveolar epithelial cells, with a 2.5-fold increase in protein levels (P < 0.05). Pin1 was localized to both the cytoplasm and nucleus. Overexpression of Pin1 led to an approximately two-fold increase in β-catenin (192%), cyclin D1 (178%), and Axin2 (165%) expression compared to controls (P < 0.01), while knockdown reduced their levels by 60%, 55%, and 63%, respectively (P < 0.01). The LMP1 overexpression increased Pin1 by 1.8-fold, strengthened its interaction with β-catenin, and amplified Wnt/β-catenin signaling. Treatment with Wnt3a further enhanced β-catenin expression by 2.4-fold, while XAV939 reduced it by 66% (P < 0.01). Pharmacological inhibition of Pin1 using Juglone and PiB significantly suppressed pathway activation, including LMP1-induced enhancement, with reductions in β-catenin levels by 68% and 72%, respectively (P < 0.01).

**Conclusions:**

Pin1 is a critical regulator of the Wnt/β-catenin pathway in PF, integrating signals from viral and cellular modulators. This study provides novel evidence of EBV-LMP1’s role in activating Pin1 in lung fibroblasts, reinforcing its value as a therapeutic target. Pin1 inhibitors effectively downregulate this signaling cascade, even under hyperactive conditions, highlighting their therapeutic potential for PF treatment. While Pin1 inhibitors effectively downregulate this signaling cascade even under hyperactive conditions, their therapeutic potential remains to be validated in preclinical models.

## 1. Background

Pulmonary fibrosis (PF) is an advanced and often deadly lung ailment characterized by the excessive accumulation of extracellular matrix, causing destruction of lung architecture and impairment of gas exchange (1). One of the key molecular pathways implicated in the disease progression of PF is the Wnt/β-catenin pathway. This cascade plays an important role in various cellular processes, including proliferation, differentiation, and survival, and its impairment is associated with several fibrotic diseases, including PF (2).

Peptidyl-prolyl cis/trans isomerase (PPIase) NIMA-interacting 1 (Pin1) has emerged as a significant regulator of the Wnt/β-catenin cascade. It has two domains: An N-terminal WW domain and a C-terminal PPIase domain (3). Pin1 is involved in the post-translational modification of proteins, influencing their stability, activity, and function. Recent studies have demonstrated that Pin1 can enhance β-catenin signaling by stabilizing β-catenin and helping it to localize to the nucleus (3).

In PF, the role of Pin1 has not been extensively studied. However, given its regulatory function in the Wnt/β-catenin pathway, investigating Pin1's expression, localization, and impact on this signaling pathway in lung fibroblasts can provide valuable insights into its potential role in PF. The Human Protein Atlas data reveals that while Pin1 expression is highest in the brain, it is also present in the lungs, suggesting its involvement in lung cellular processes (4).

Viral factors such as the Epstein-Barr virus (EBV)-encoded latent membrane protein 1 (LMP1) have been shown to interact with cellular signaling pathways, including Wnt/β-catenin, in various pathological contexts (5). The LMP1, a well-established EBV oncoprotein, mimics tumor necrosis factor receptor signaling to modulate cellular pathways critical for proliferation, survival, and inflammation (6). The LMP1 can amplify β-catenin signaling, as suggested by its role in various cancers, including nasopharyngeal carcinoma and Hodgkin's lymphoma (7). While LMP1’s oncogenic effects are well documented, its ability to amplify β-catenin signaling may also contribute to fibrosis-related mechanisms. These findings underscore the potential for viral factors like LMP1 to exacerbate aberrant signaling cascades relevant to PF.

## 2. Objectives

In this study, we aim to characterize the expression and localization of Pin1 in various cell lines, including human lung fibroblasts (MRC-5) and cancer cell lines (A549, H1299), to determine the most appropriate model for studying PF. Furthermore, we investigate the effects of Pin1 modulation through overexpression and inhibition on the Wnt/β-catenin pathway in MRC-5 cells. We also explore the impact of Wnt pathway activators (Wnt3a) and inhibitors (XAV939) on Pin1 and β-catenin. Finally, we assess the effects of Pin1 inhibitors, such as Juglone and PiB, on the Wnt/β-catenin cascade to evaluate their potential as therapeutic agents in modulating this pathway in PF.

By incorporating EBV-LMP1 into our experimental framework, we also examine its role in regulating Pin1 and β-catenin activity, providing a unique perspective on the interplay between viral and cellular factors in disease progression. This comprehensive approach will offer novel insights into the function of Pin1 in PF and its potential as a therapeutic target. The findings from this study could pave the way for developing new treatment strategies for PF by targeting the Pin1-mediated modulation of the Wnt/β-catenin pathway.

## 3. Methods

### 3.1. Plasmid Construction

The pcDNA3- wild-type Pin1 (Pin1 WT) expression vector and LMP1 expression vector were constructed as described previously (8, 9).

### 3.2. Cell Culture

Human lung fibroblasts (MRC-5), HepG2 (hepatoblastoma cell line), HEK 293T (human embryonic kidney cells), alveolar epithelial type II cells (AEII), and SH-SY5Y (human neuroblastoma cells) were cultured in Dulbecco's Modified Eagle Medium (DMEM) as described previously (9). H1299 (human non-small cell lung cancer) and A549 (human lung carcinoma) cells were cultured in RPMI-1640 supplemented with 10% FBS and 1% penicillin-streptomycin. Subculturing was performed every 3 days for all cell lines, except for HEK 293T cells, which were subcultured every 2 days.

### 3.3. DNA Transfection

MRC-5 cells were transfected with Pin1 siRNA (Santa Cruz #sc-36230), negative-control siRNA (Invitrogen #4390843), LMP1 construct, Pin1 WT construct, or the pcDNA3 vector. Transfections were carried out with Lipofectamine 2000 (Invitrogen) following the manufacturer's guidelines. For siRNA transfection, 100 pmol of siRNA and 4 µL of Lipofectamine 2000 were each diluted in 200 µL of Opti-MEM (Gibco), combined, and incubated for 20 minutes at room temperature to form siRNA-lipid complexes. For DNA transfections, 4 µg of either LMP1, Pin1 WT construct, or pcDNA3 and 4 µL of Lipofectamine 2000 were similarly diluted, combined, and incubated as described previously (10).

Mock MRC-5 cells were used as the negative control for all transfection experiments. These cells were subjected to the same transfection conditions without the introduction of plasmid or siRNA, ensuring that any observed effects were specifically due to the introduced constructs rather than the transfection procedure itself.

### 3.4. Western Blotting

Cells were lysed, and membranes were prepared as previously described (10, 11). Primary antibodies used in this study included Pin1 (Abcam, #ab53361), β-catenin (cell signaling technology, #8480), cyclin D1 (Abcam, #ab16663), Axin2 (cell signaling technology, #2151), and GAPDH (loading control, cell signaling technology, #5174). The catalog numbers of all primary antibodies have been specified to ensure reproducibility. The membranes were subsequently incubated with HRP-conjugated secondary antibodies, and densitometric analysis was performed as done previously. Densitometric analysis was performed using ImageJ. For each protein, signal intensity was normalized to GAPDH. The expression level in mock-transfected cells was set as 100%, and all treatment groups were expressed relative to this baseline.

### 3.5. Cytoplasmic and Nuclear Fractionation

MRC-5 cells were transfected with 4 µg of either pcDNA3 or Pin1 WT, and cytoplasmic and nuclear fractions were then prepared. Cell lysates were prepared using cold harvest buffer [0.5% Triton X-100, 10 mM HEPES (pH 7.9), 0.5 M sucrose, 50 mM NaCl, 10 mM NaF, 0.1 mM EDTA, 1 mM dithiothreitol (DTT)]. The lysates were incubated on ice for 5 minutes to allow lysis. Subsequently, the samples were centrifuged in a swinging-bucket rotor at 100 × g for 10 minutes. The supernatant was collected as the cytoplasmic fraction, while the pellet, containing the nuclear components, was carefully resuspended (12, 13). The purity of the fractions was verified using GAPDH as a cytoplasmic marker and histone H3 as a nuclear marker.

### 3.6. Co-immunoprecipitation

MRC-5 cells were transfected with the LMP1 construct, and cells were harvested 3 days post-transfection. To assess the physical interaction between Pin1 and β-catenin, cell lysates were immunoprecipitated using Pin1 antibodies, followed by immunoblotting with β-catenin antibodies, according to previously established protocols. As a negative control for non-specific binding, parallel immunoprecipitations were performed using species- and isotype-matched control IgG, serving as the isotype control (12, 13).

### 3.7. Juglone and PiB Treatments

To examine the effects of Pin1 inhibitors on PF, MRC-5 cells were treated with juglone (Sigma-Aldrich; AG17724) dissolved in ethanol or PiB (Calbiochem; CAS 64005-90-9) dissolved in DMSO as defined previously (9). The effective concentrations (20 µM) were selected based on a thorough literature review and preliminary dose-response experiments. MTT assays were performed to ensure that the selected concentrations produced significant pathway modulation without causing cytotoxicity. Dose-response analysis revealed that the IC_50_ values for inhibiting β-catenin expression were approximately 18.5 µM for Juglone and 21.3 µM for PiB, justifying the choice of 20 µM as the optimal working concentration.

### 3.8. Wnt3a and XAV939 Treatments

To investigate the influence of Wnt effects on PF, MRC-5 cells were treated with Wnt3a and XAV939. Wnt3a (R&D Systems, Cat# 5036-WN-025/CF) was reconstituted in sterile PBS to a concentration of 50 ng/mL. XAV939 (Sigma-Aldrich, Cat# X3004) was dissolved in DMSO to prepare a stock solution of 10 mM. MRC-5 cells were seeded in 6-cm plates and allowed to reach 70 - 80% confluency. MRC-5 cells were transfected with pcDNA3 or Pin1 WT constructs, and 48 hours after transfections, the cells were treated either with 50 ng/mL of Wnt3a or vehicle (PBS), XAV939 (20 μM), or an equivalent volume of DMSO as a control for 24 hours. The effective concentration of Wnt3a was determined through literature review and preliminary dose-response analysis, selecting the dose that maximized pathway activation while maintaining cell viability.

### 3.9. Statistical Analysis

Each experiment was repeated at least three times, and results were expressed as mean ± standard deviation. Statistical analysis was performed using Student’s *t*-test (GraphPad Prism, version 7.0). For multiple comparisons, Bonferroni correction was applied to minimize type I errors. Significance levels were denoted as * P < 0.05, ** P < 0.01, and *** P < 0.001. Images were analyzed using ImageJ software.

## 4. Results

### 4.1. Characterization of Pin1 Expression and Localization in Pulmonary Fibrosis Cells

**Figure 1.**
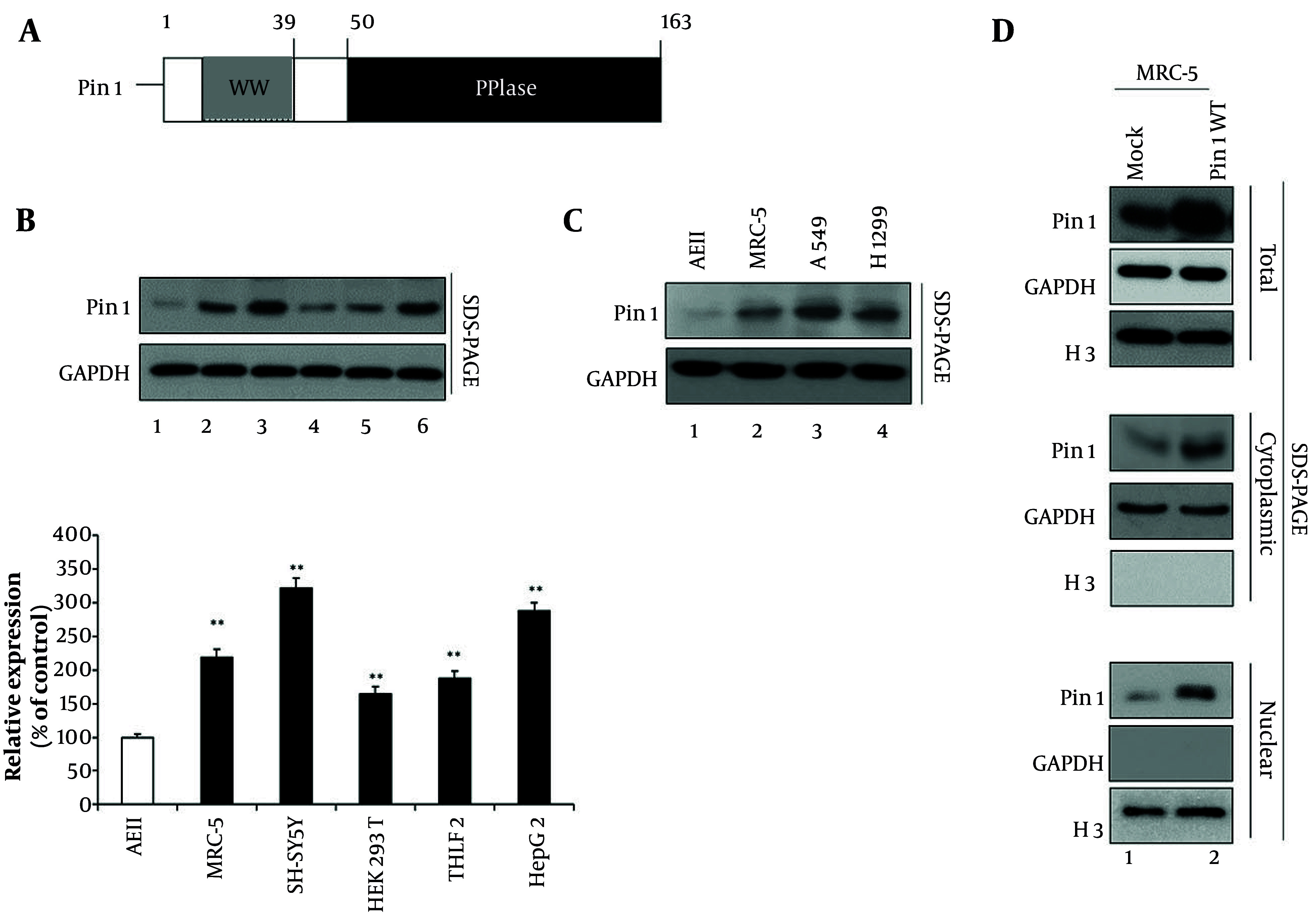
Structural and expression analysis of Pin1 in pulmonary fibrosis (PF) cells. A, schematic representation of the Pin1 protein, showing its WW domain at the N-terminal and peptidyl-prolyl cis/trans isomerase (PPIase) domain at the C-terminal end. This structural organization is essential for Pin1's function in the post-translational modification of proteins. B, comparative analysis of endogenous Pin1 expression in various cell lines. Western blotting was performed using a Pin1 antibody on AEII cells, MRC-5 cells, SH-SY5Y cells, HEK 293T cells, THLE2 cells, and HepG2 cells (lanes 1 - 6). GAPDH served as the loading control. Densitometric quantification from three independent experiments is presented in the lower panel as a column chart, showing relative expression values (100, 218, 321, 165, 187, and 287) with mean ± standard deviation (SD). All increases were statistically significant (P < 0.01) compared to the control. C, comparative Pin1 expression in human lung fibroblast cells versus cancer cells. Western blotting was performed using a Pin1 antibody on AEII cells, A549, and H1299 cancer cell lines (lanes 1 - 3). D, subcellular localization of Pin1 in MRC-5 cells. Cytoplasmic and nuclear fractions of mock or wild-type Pin1 (Pin1 WT) transfected MRC-5 cells were prepared. Total (panels 1 - 3), cytoplasmic (lanes 4 - 6), and nuclear (lanes 7 - 9) fractions were visualized using specific antibodies against Pin1. GAPDH was used as the cytoplasmic marker and H3 as the nuclear fraction marker. (** P < 0.01)

Pin1 plays a role in the post-translational modification of proteins (3, 9). Pin1 has an N-terminal WW domain and a C-terminal PPIase domain (Figure 1A). Based on information from the Human Protein Atlas, Pin1 protein expression is highest in the brain, with an RNA expression level of 259.9 transcripts per million (TPM) (3). In comparison, the RNA expression level of Pin1 in lung tissue is lower, approximately 40.4 TPM. Moreover, we conducted a comparative analysis of endogenous Pin1 expression in various cell lines, including AEII, MRC-5, SH-SY5Y, HEK 293T, THLE2, and HepG2 (Figure 1B). Our findings indicate that MRC-5 cells exhibit significantly higher Pin1 expression compared to AEII cells, demonstrating a clear superiority in terms of Pin1 protein levels (Figure 1 lane 1 vs. 2), with SH-SY5Y showing the highest Pin1 expression (Figure 1 lane 1 and 2 vs. 3). Additionally, HepG2 cells also demonstrate elevated Pin1 expression compared to THLE2 cells (Figure 1 lane 5 vs. 6).

In addition, densitometric quantification of Western blots from three independent experiments is presented in the lower panel of Figure 1B. The relative expression values were 100, 218, 321, 165, 187, and 287 across the tested conditions, with all increases showing statistical significance (P < 0.01) compared to the control. The rationale for using these cell lines lies in comparing Pin1 expression across diverse tissue origins. MRC-5 cells were chosen to model PF as they represent lung fibroblasts, while AEII cells provide an epithelial lung cell comparison. SH-SY5Y cells, representing neural tissue, were included due to the high brain expression of Pin1. HepG2 and THLE2 cells represent liver tissue to evaluate hepatic Pin1 expression, and HEK 293T cells were used as a human kidney cell reference. This comparative approach allows a comprehensive analysis of Pin1 expression across different tissue types, highlighting its variable expression profile.

Subsequently, we assessed which cell line would be most appropriate for studying PF, considering human lung fibroblast cells versus cancer cells such as A549 or H1299 (Figure 1C). MRC-5 cells were chosen as the optimal model for PF studies due to their significantly higher Pin1 expression compared to AEII cells, alongside their normal lung fibroblast characteristics, which better represent fibrotic conditions compared to epithelial cancer cell lines. A549 or H1299 cells exhibited the highest Pin1 expression (Figure 1 lane 1 and 2 vs. 3 and 4). We opted for MRC-5 human lung fibroblast cells for further experiments due to their appropriateness; these cells are derived from normal lung tissue and are commonly used to model fibroblast behavior in fibrotic conditions. Cancer cell lines, such as A549 or H1299, may not be suitable for studying PF as they do not accurately represent the behavior of fibroblasts in fibrotic conditions, given their epithelial cell origin and different signaling and proliferation characteristics.

Furthermore, we investigated the subcellular localization of Pin1 by fractionating mock or Pin1 WT transfected MRC-5 cells (Figure 1D). Our analysis revealed that Pin1 protein is distinctly present in both the cytoplasmic and nuclear fractions, indicating its dual localization within the cell. The purity of the fractions was confirmed using GAPDH as a cytoplasmic marker and H3 as a nuclear marker, ensuring accurate subcellular fractionation. This dual localization suggests that Pin1 may exert its biological functions in both cytoplasmic and nuclear compartments, thereby potentially influencing a broad spectrum of cellular processes associated with PF pathogenesis.

### 4.2. Pin1 and Latent Membrane Protein 1 Cooperatively Enhance Wnt/β-Catenin Signaling

**Figure 2.**
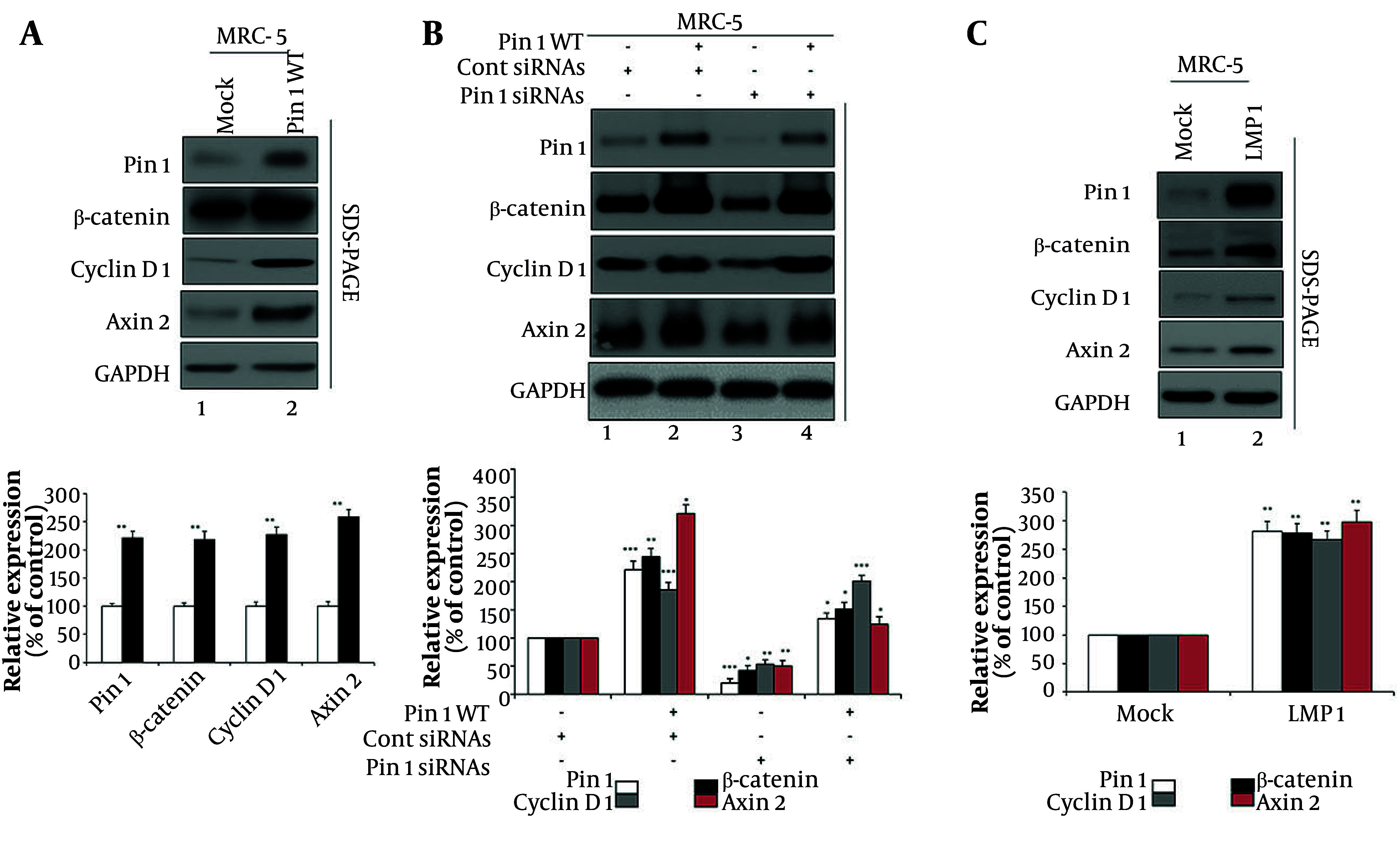
Pin1 and latent membrane protein 1 (LMP1) modulation of Wnt/β-catenin signaling in MRC-5 cells: A, Pin1 overexpression enhances Wnt/β-catenin signaling. MRC-5 cells were mock-transfected or transfected with 4 µg wild-type Pin1 (Pin1 WT). Seventy-two hours post-transfection, cells were lysed for analysis. Densitometric quantification showed that Pin1 WT increased β-catenin (218%), Cyclin D1 (227%), and Axin2 (258%) relative to mock (100%, P < 0.01). B, Pin1 knockdown reduces β-catenin signaling (lane 1: Untreated MRC-5 cells; lane 2: Control siRNAs and 4 µg of Pin1 WT; lane 3: Pin1-specific siRNAs; lane 4: The Pin1 WT overexpression with Pin1-specific siRNAs). Quantification showed Pin1 at 222% with WT, suppressed to 21% with siRNA, and restored to 135% with WT+siRNA. β-catenin rose to 245%, dropped to 43%, and recovered to 152%. Cyclin D1 increased to 186%, decreased to 54%, and restored to 201%. Axin2 surged to 321%, reduced to 51%, and partially restored to 125%. All changes were statistically significant (P < 0.05 to P < 0.001). C, LMP1 expression modulates Wnt/β-catenin signaling. MRC-5 cells were mock-transfected or transfected with 4 µg LMP1. Cells were lysed 72 hours post-transfection. Quantification confirmed that LMP1 significantly increased Pin1 (281%), β-catenin (278%), cyclin D1 (267%), and Axin2 (298%) relative to mock (100%, P < 0.01). Negative (-) and positive (+) signs: The negative (-) sign indicates the absence of a specific treatment (e.g., no siRNA or overexpression), while the positive (+) sign indicates the presence of the respective treatment (e.g., Pin1 WT or siRNA). Primary antibodies: Pin1 (Abcam, #ab53361), β-catenin (cell signaling technology, #8480), cyclin D1 (Abcam, #ab16663), Axin2 (cell signaling technology, #2151), and GAPDH (loading control, cell signaling technology, #5174). Relative levels of Pin1, β-catenin, cyclin D1, and Axin2 were quantified using ImageJ 1.46 (data represent mean values from three separate experiments; * P < 0.05, ** P < 0.01, and *** P < 0.001).

To explore the role of Pin1 in regulating the Wnt/β-catenin pathway, which is crucial for various cellular processes (14, 15), we first assessed the effects of Pin1 overexpression (Figure 2A). Overexpression of Pin1 led to an approximately two-fold activation of the signaling pathway, as indicated by increased expression levels of β-catenin, cyclin D1, and Axin2 (Figure 2 lane 1 vs. lane 2). Quantitative densitometry analysis confirmed that Pin1 overexpression significantly enhanced the expression of β-catenin, cyclin D1, and Axin2 compared to the Mock group (Figure 2A, lower panel). Relative expression levels increased approximately two-fold, with statistical significance (P < 0.01), supporting the activation of the Wnt/β-catenin signaling cascade.

Next, we checked the effects of Pin1 knockdown on β-catenin signaling (Figure 2B). Four conditions were tested: Untreated control (lane 1), control siRNAs (lane 2), Pin1 siRNAs (lane 3), and Pin1 WT along with Pin1 siRNAs (lane 4). The Western blot analysis indicates that Pin1 expression significantly affects β-catenin, cyclin D1, and Axin2 proteins in MRC-5 cells. Silencing Pin1 (lane 3) drastically reduces the expression of these proteins, while overexpressing Pin1 WT (lane 4) can partially restore their levels. The observed changes in protein expression suggest that Pin1 positively regulates β-catenin, cyclin D1, and Axin2, and its knockdown results in their decreased expression, potentially implicating Pin1 in the regulation of pathways involving these proteins. Quantitative densitometry (Figure 2B, lower panel) showed that Pin1 expression itself increased to 222% (P < 0.001) with WT plus control siRNA, was suppressed to 21% (P < 0.001) with Pin1 siRNA, and partially restored to 135% (P < 0.05) with WT+siRNA. The β-catenin rose to 245% (P < 0.01), dropped to 43% (P < 0.05), and was restored to 152% (P < 0.05) under the same conditions. Cyclin D1 increased to 186% (P < 0.001), decreased to 54% (P < 0.01), and recovered to 201% (P < 0.001). Similarly, Axin2 levels surged to 321% (P < 0.05), were reduced to 51% (P < 0.01), and partially restored to 125% (P < 0.05). These quantitative data further validate the regulatory role of Pin1 in modulating β-catenin pathway components.

Additionally, we examined the effect of LMP1 expression on this pathway (Figure 2C). Western blot analysis of mock- and LMP1-transfected MRC-5 cells revealed that LMP1 significantly upregulated the expression of Pin1, β-catenin, cyclin D1, and Axin2 compared to mock-transfected cells (Figure 2 lane 1 vs. 2). Quantitative analysis (Figure 2C, lower panel) confirmed that LMP1 transfection markedly increased Pin1 (281%), β-catenin (278%), cyclin D1 (267%), and Axin2 (298%) compared to mock cells (P < 0.01 for all). Taken together, these results establish that Pin1 is a key regulator of β-catenin signaling and its downstream targets, while LMP1 enhances the pathway by upregulating Pin1 and related proteins, suggesting a potential link between viral factors and the activation of this signaling cascade.

### 4.3. Pin1 Synergizes with Wnt3a and Latent Membrane Protein 1 to Modulate the Wnt/β-Catenin Pathway in Pulmonary Fibrosis

**Figure 3.**
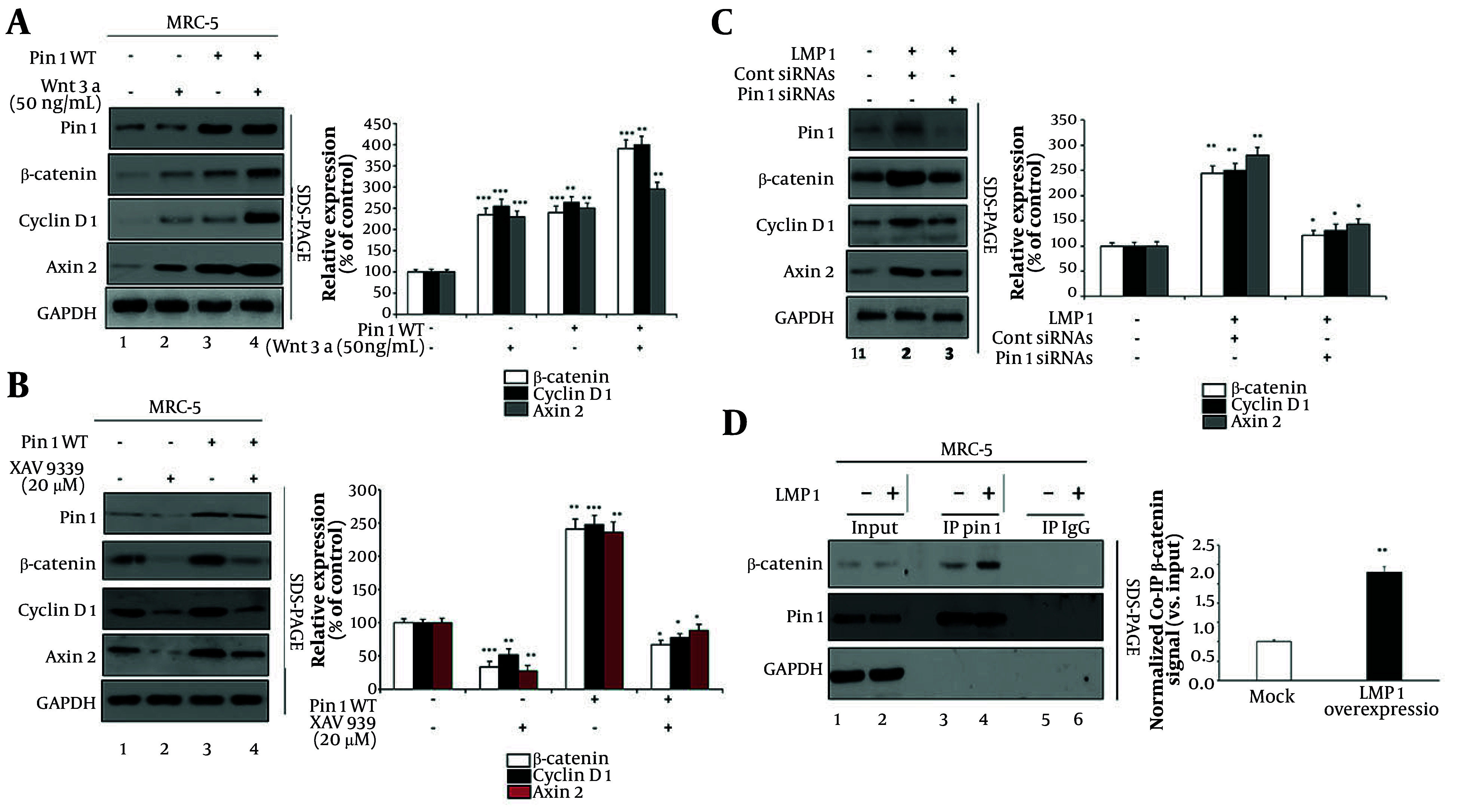
Effects of Wnt signaling modulators on Pin1 and β-catenin signaling: A, effects of Wnt3a and Pin1 overexpression on β-catenin components. Cells were treated under four conditions: Untreated control (lane 1), Wnt3a treatment (50 ng/mL) (lane 2), wild-type Pin1 (Pin1 WT, lane 3), and Pin1 WT overexpression with Wnt3a treatment (lane 4). Densitometric analysis showed that β-catenin increased from 100% in control to 236% (P < 0.001) with Wnt3a, 241% (P < 0.001) with Pin1 WT, and 392% (P < 0.001) with Wnt3a + Pin1 WT. Cyclin D1 rose to 256% (P < 0.001), 264% (P < 0.01), and 401% (P < 0.01), while Axin2 reached 231% (P < 0.001), 251% (P < 0.01), and 295% (P < 0.01), respectively. B, effects of XAV939 and Pin1 overexpression on β-catenin signaling components. Cells were treated under four conditions: Untreated control (lane 1), XAV939 treatment (20 µM) (lane 2), Pin1 WT overexpression (lane 3), and Pin1 WT overexpression with XAV939 treatment (lane 4). Quantitative densitometry revealed that β-catenin decreased to 34% (P < 0.001) with XAV939, increased to 253% (P < 0.01) with Pin1 WT, and was partly restored to 67% (P < 0.05) with XAV939+Pin1 WT. Cyclin D1 fell to 52% (P < 0.01), rose to 248% (P < 0.001), and was restored to 78% (P < 0.01). Axin2 decreased to 28% (P < 0.01), increased to 236% (P < 0.01), and was restored to 89% (P < 0.05). C, effects of latent membrane protein 1 (LMP1) and Pin1 knockdown on Wnt/β-catenin signaling in MRC-5 cells. Lane 1 represents mock-transfected cells, lane 2 shows cells transfected with LMP1 and treated with control siRNAs, and lane 3 depicts cells transfected with LMP1 and treated with Pin1-specific siRNAs. Densitometric quantification confirmed that LMP1 significantly increased β-catenin to 244% (P < 0.01), cyclin D1 to 250% (P < 0.01), and Axin2 to 280% (P < 0.01). Silencing Pin1 in the presence of LMP1 reduced β-catenin to 121% (P < 0.05), cyclin D1 to 131% (P < 0.05), and Axin2 to 143% (P < 0.05). D, co-immunoprecipitation (Co-IP) analysis showing interaction between Pin1 and β-catenin pathway proteins. Lanes 1 and 2 represent input samples, lanes 3 and 4 show Co-IP using a Pin1 antibody, and lanes 5 and 6 represent Co-IP using IgG as the control. Antibodies used were Pin1 (Abcam, #ab53361), β-catenin (cell signaling technology, #8480), cyclin D1 (Abcam, #ab16663), Axin2 (cell signaling technology, #2151), and GAPDH (loading control, cell signaling technology, #5174). Relative levels of Pin1, β-catenin, cyclin D1, and Axin2 were quantified using ImageJ 1.46r (data is shown as the mean values from three separate experiments. Statistical significance was determined by Student's *t*-test; significance levels are * P < 0.05, ** P < 0.01, and *** P < 0.001 compared to the corresponding controls; ‘+’ and ‘−’ the presence or absence of specific treatments such as Wnt3a, XAV939, Pin1 WT overexpression, LMP1, or siRNA, as described in each panel).

To elucidate the role of Pin1 in modulating Wnt/β-catenin signaling under various conditions, we evaluated the effects of Wnt signaling activators and inhibitors, as well as LMP1 expression, on this pathway. First, we assessed the effects of Wnt3a and Pin1 overexpression on β-catenin signaling (Figure 3A). MRC-5 cells were treated under four conditions: Untreated control (lane 1), Wnt3a (50 ng/mL) (lane 2), Pin1 overexpression (Pin1 WT, lane 3), and Pin1 overexpression combined with Wnt3a treatment (lane 4). Wnt3a treatment alone significantly enhanced the expression of β-catenin (236%), cyclin D1 (256%), and Axin2 (231%) compared to the control (lane 1 vs. 2). Similarly, Pin1 overexpression alone increased the expression of these proteins (lane 1 vs. 3). Importantly, combining Wnt3a treatment with Pin1 overexpression resulted in the highest expression levels of β-catenin (392%), cyclin D1 (401%), and Axin2 (295%), demonstrating a synergistic effect (lane 2 vs. 4 and lane 3 vs. 4).

The side panel (3A) shows quantitative densitometry confirming that β-catenin increased from 100% in control to 236% (P < 0.001) with Wnt3a, 241% (P < 0.001) with Pin1 WT, and 392% (P < 0.001) with Wnt3a+Pin1 WT. Cyclin D1 rose to 256% (P < 0.001), 264% (P < 0.01), and 401% (P < 0.01) under the same conditions. Likewise, Axin2 levels were elevated to 231% (P < 0.001), 251% (P < 0.01), and 295% (P < 0.01), respectively.

Next, we investigated the effects of the Wnt signaling inhibitor XAV939 in the context of Pin1 overexpression (Figure 3B). MRC-5 cells were treated under four conditions: Untreated control (lane 1), XAV939 (20 µM, lane 2), Pin1 overexpression (Pin1 WT) (lane 3), and Pin1 overexpression combined with XAV939 treatment (lane 4). XAV939 treatment alone significantly reduced the expression of β-catenin (34%), cyclin D1 (52%), and Axin2 (236%) compared to the control (lane 1 vs. 2). While Pin1 overexpression amplified the expression of these proteins (β-catenin 253%, cyclin D1 248%, Axin2 236%; lane 1 vs. 3), co-treatment with XAV939 and Pin1 overexpression moderately restored their levels compared to XAV939 treatment alone (β-catenin 67%, cyclin D1 78%, Axin2 89%; lane 2 vs. 4). These results indicate that Pin1 can partially counteract the inhibitory effects of XAV939 on Wnt/β-catenin signaling.

Quantitative densitometry (Figure 3B, side panel) revealed that β-catenin decreased from 100% in control to 34% (P < 0.001) with Pin1 knockdown, while Pin1 WT overexpression raised it to 241% (P < 0.01) and co-transfection with siRNA reduced it to 67% (P < 0.05). Similarly, cyclin D1 dropped to 52% (P < 0.01) with knockdown, increased to 248% (P < 0.001) with Pin1 WT, and was partly restored to 78% (P < 0.01) in the co-treatment. Axin2 followed the same trend, decreasing to 28% (P < 0.01), rising to 236% (P < 0.01), and falling to 89% (P < 0.05) under respective conditions.

To further understand the role of Pin1 in Wnt/β-catenin regulation, we evaluated the effect of LMP1 expression (Figure 3C). MRC-5 cells were treated with mock transfection (lane 1), LMP1 plus control siRNA (lane 2), and LMP1 plus Pin1 siRNA (lane 3). The LMP1 expression significantly enhanced the levels of β-catenin, cyclin D1, and Axin2 (lane 1 vs. 2). However, Pin1 knockdown in the presence of LMP1 markedly reduced the expression of these proteins (lane 2 vs. 3), demonstrating that Pin1 is essential for the upregulation of the Wnt/β-catenin pathway mediated by LMP1.

Quantitative densitometry (Figure 3C, side panel) showed that LMP1 expression significantly increased β-catenin to 244% (P < 0.01) and cyclin D1 to 250% (P < 0.01), along with Axin2 reaching 280% (P < 0.01) compared to control. However, silencing Pin1 in the presence of LMP1 reduced β-catenin to 121% (P < 0.05), cyclin D1 to 131% (P < 0.05), and Axin2 to 143% (P < 0.05), indicating that Pin1 is required for the full LMP1-mediated upregulation of Wnt/β-catenin signaling.

Lastly, co-immunoprecipitation (Co-IP) experiments revealed that Pin1 physically interacts with β-catenin, and this interaction is significantly enhanced in LMP1-overexpressing cells compared to mock-transfected cells (Figure 3 lane 3 vs. 4). Densitometric quantification of the Co-IP bands, normalized to input, confirmed a substantial increase in the β-catenin interaction signal in LMP1-expressing cells. This finding highlights a mechanistic link between LMP1 expression and the activation of Wnt/β-catenin signaling via Pin1.

It is important to clarify that our study did not specifically investigate whether LMP1 directly binds to Pin1. Instead, we assessed the enhancement of the Pin1/β-catenin interaction in the presence of LMP1 overexpression. The increased interaction observed in Figure 3D is likely due to LMP1-mediated upregulation of Pin1 expression rather than a direct physical interaction between LMP1 and Pin1. Future studies will include Co-IP experiments using LMP1-specific antibodies to explore any potential direct binding between LMP1 and Pin1.

Overall, these results indicate that Pin1 is a critical regulator of the Wnt/β-catenin pathway, with its activity being modulated by Wnt3a, XAV939, and LMP1. The LMP1 enhances both Pin1 expression and its interaction with β-catenin, contributing to the activation of the pathway, while Pin1 plays a central role in integrating upstream signals to regulate downstream targets.

### 4.4. Therapeutic Potential of Pin1 Inhibitors in Modulating the Wnt/β-Catenin Cascade in Pulmonary Fibrosis

**Figure 4.**
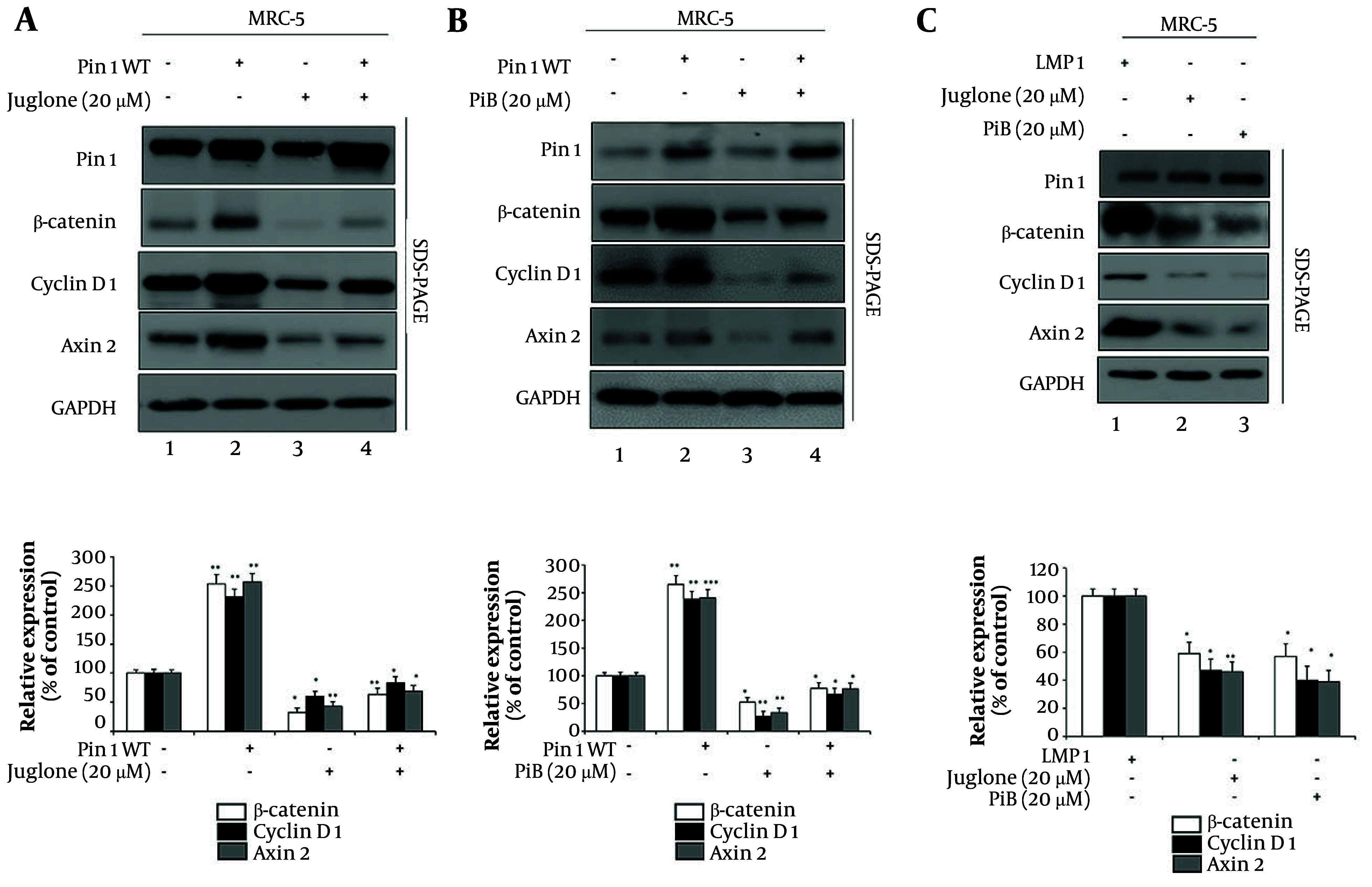
Effects of Pin1 inhibitors on Wnt/β-catenin pathway: A, effects of Juglone on Wnt/β-catenin signaling components. MRC-5 cells were treated under four conditions: Untreated control (lane 1), Juglone treatment (20 µM, lane 2), wild-type Pin1 (Pin1 WT) overexpression (lane 3), and Pin1 WT overexpression combined with Juglone treatment (lane 4). Juglone, as a Pin1 inhibitor, primarily reduces Pin1 activity rather than its protein levels. Therefore, the presence of Pin1 protein in lane 2 does not indicate the absence of inhibition but reflects that the inhibitor does not decrease total protein levels. Densitometric quantification (lower panel) confirmed significant reductions of β-catenin, cyclin D1, and Axin2 upon Juglone treatment compared to Pin1 WT (P < 0.05 - 0.01). B, effects of PiB on Wnt/β-catenin cascade components. MRC-5 cells were treated under similar conditions as in (A): Untreated control (lane 1), PiB treatment (20 µM, lane 2), Pin1 WT overexpression (lane 3), and Pin1 WT overexpression with PiB treatment (lane 4). Similar to Juglone, PiB inhibits Pin1 activity without significantly reducing total Pin1 protein levels. Quantitative analysis (lower panel) showed that PiB markedly suppressed Pin1-induced increases in β-catenin, cyclin D1, and Axin2 (P < 0.05 - 0.001). C, effects of latent membrane protein 1 (LMP1), Juglone, and PiB on Wnt/β-catenin signaling. Lane 1 shows cells transfected with LMP1 alone, lane 2 depicts cells transfected with LMP1 and treated with Juglone (20 µM), and lane 3 represents cells transfected with LMP1 and treated with PiB (20 µM). The LMP1 alone enhances Wnt/β-catenin signaling, while co-treatment with Pin1 inhibitors reduces pathway activation, demonstrating the ability of Juglone and PiB to attenuate LMP1-mediated enhancement of the signaling cascade. Densitometry (lower panel) demonstrated that both Juglone and PiB significantly reduced LMP1-induced upregulation of β-catenin, cyclin D1, and Axin2 [P < 0.05; ‘+’ the inclusion of a specific treatment (e.g., Juglone, PiB, Pin1 WT, LMP1), ‘−’ its absence, in all panels] (* P < 0.05, ** P < 0.01, and *** P < 0.001).

To evaluate the role of Pin1 inhibitors, Juglone and PiB, in regulating the Wnt/β-catenin signaling pathway, we analyzed their effects in MRC-5 cells under various conditions (Figure 4). First, we examined the impact of Juglone on Pin1-mediated modulation of the Wnt/β-catenin pathway (Figure 4A). MRC-5 cells were treated under four conditions: Untreated control (lane 1), Juglone (20 µM) (lane 2), Pin1 overexpression (Pin1 WT, lane 3), and Pin1 overexpression combined with Juglone treatment (lane 4). Pin1 overexpression significantly elevated the expression of β-catenin (254%), cyclin D1 (231%), and Axin2 (257%) compared to the control (lane 1 vs. 2), demonstrating that Pin1 enhances the Wnt/β-catenin cascade. However, Juglone treatment alone resulted in a marked reduction in β-catenin (32%), cyclin D1 (60%), and Axin2 (43%) levels (lane 1 vs. 3), indicating potent suppression of the pathway. Interestingly, co-treatment with Juglone and Pin1 overexpression partially reversed the effects of Pin1 overexpression, with β-catenin (64%), cyclin D1 (85%), and Axin2 (69%) significantly reduced compared to Pin1 overexpression alone (lane 2 and 3 vs. 4).

To support dose selection, we conducted MTT-based cytotoxicity assays for Juglone, PiB, and Wnt3a, and selected doses that maintained ≥ 80% cell viability in MRC-5 cells ((Table S1 can be found in Supplementary File). Quantitative densitometry (Figure 4A, lower panel) confirmed that β-catenin was 254% (P < 0.01) with Pin1 WT, suppressed to 32% (P < 0.05) by Juglone, and reduced to 64% (P < 0.01) with Juglone+Pin1 WT compared to Pin1 WT alone. Cyclin D1 rose to 231% (P < 0.01) with Pin1 WT, decreased to 60% (P < 0.05) with Juglone, and was partly restored to 83% (P < 0.05) under co-treatment. Similarly, Axin2 increased to 257% (P < 0.01) with Pin1 WT, but dropped to 43% (P < 0.01) with Juglone and 69% (P < 0.01) under co-treatment.

A similar trend was observed with PiB (Figure 4B). PiB treatment effectively suppressed β-catenin signaling, counteracting the activation induced by Pin1 overexpression. These findings confirm that both Juglone and PiB can effectively inhibit the Wnt/β-catenin pathway, even in the presence of enhanced Pin1 activity. Quantitative densitometry (Figure 4B, lower panel) showed that β-catenin increased to 265% (P < 0.01) with Pin1 WT, was reduced to 53% (P < 0.05) with PiB, and remained suppressed at 78% (P < 0.05) with PiB+Pin1 WT compared to Pin1 WT alone. Cyclin D1 rose to 239% (P < 0.01) under Pin1 WT, but dropped to 27% (P < 0.05) with PiB and 67% (P < 0.05) with PiB+Pin1 WT. Similarly, Axin2 expression reached 241% (P < 0.001) with Pin1 WT, decreased to 34% (P < 0.05) under PiB, and remained suppressed at 77% (P < 0.05) with co-treatment. These results confirm that PiB strongly counteracts Pin1-mediated activation of the Wnt/β-catenin pathway.

Additionally, we investigated the effects of Juglone and PiB in the context of LMP1-mediated pathway activation (Figure 4C). The LMP1 expression alone increased β-catenin, cyclin D1, and Axin2 (lane 1). However, treatment with either Juglone or PiB in LMP1-expressing cells markedly downregulated the expression of these proteins, effectively countering the pathway activation induced by LMP1 (lane 1 vs. 2 and 3). Quantitative densitometry (Figure 4C, lower panel) confirmed that LMP1 expression alone maintained baseline levels of β-catenin, cyclin D1, and Axin2 at 100%. Treatment with Juglone significantly reduced β-catenin to 59% (P < 0.05), cyclin D1 to 47% (P < 0.05), and Axin2 to 46% (P < 0.05). Similarly, PiB treatment decreased β-catenin to 57% (P < 0.05), cyclin D1 to 40% (P < 0.05), and Axin2 to 39% (P < 0.05). These results demonstrate that both Juglone and PiB effectively counteract LMP1-induced activation of Wnt/β-catenin signaling, further validating Pin1 as a critical target for suppressing viral-mediated pathway activation.

Together, these findings highlight the dual regulatory role of Pin1 in the Wnt/β-catenin pathway, where its overexpression enhances signaling while its inhibition by Juglone or PiB significantly suppresses pathway activity. The ability of Pin1 inhibitors to downregulate the Wnt/β-catenin cascade, even in the context of strong activators like LMP1, underscores their therapeutic potential in diseases associated with aberrant Wnt/β-catenin signaling, such as PF.

### 4.5. Proposed Model: Regulation of the Wnt/β-Catenin Pathway by Pin1 and Its Modulators

**Figure 5.**
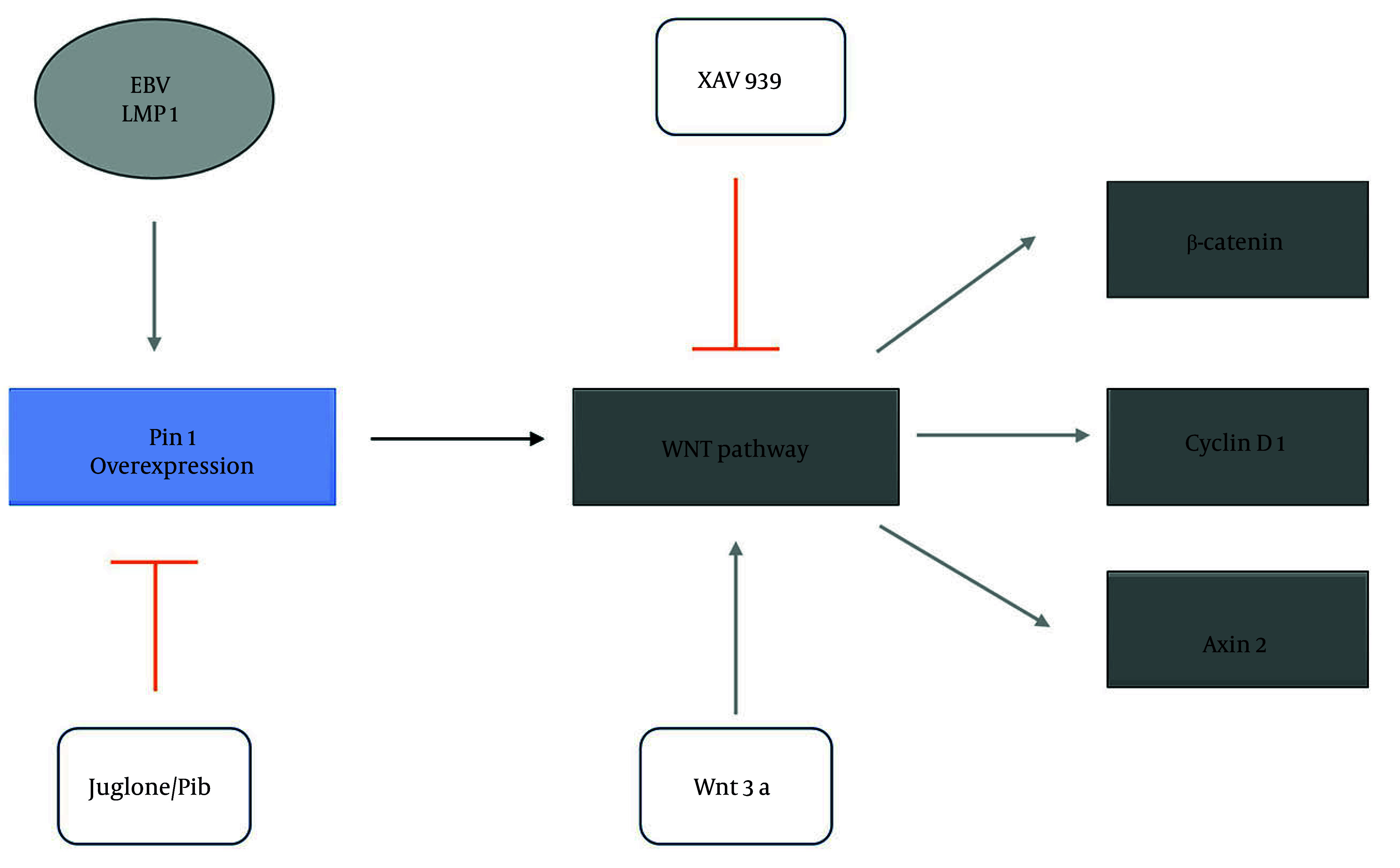
Schematic representation of Pin1 modulation in the Wnt/β-catenin pathway and its regulation by Epstein-Barr virus (EBV)-latent membrane protein 1 (LMP1), inhibitors, and implications in pulmonary fibrosis (PF): A schematic diagram illustrating the regulatory effects of Pin1 on the Wnt/β-catenin signaling pathway. Arrowheads indicate activation or promotion of signaling, while bar-ended arrows represent inhibition. Pin1 overexpression (Pin1 box) activates the Wnt/β-catenin pathway, leading to increased levels of β-catenin and its downstream targets cyclin D1 and Axin2. The EBV-LMP1 (EBV LMP1 oval) enhances Pin1 expression, further promoting the pathway. Inhibition of the Wnt pathway is represented by XAV939 (bar-ended arrow), which suppresses β-catenin stabilization. Juglone and PiB (bar-ended arrows) inhibit Pin1 activity, reducing pathway activation.

The schematic model (Figure 5) illustrates the regulatory dynamics of Pin1 in the Wnt/β-catenin signaling pathway. Activation occurs via Pin1 overexpression, which enhances β-catenin stabilization and upregulates its downstream targets, cyclin D1 and Axin2, a process further amplified by EBV-LMP1. The LMP1 also strengthens the interaction between Pin1 and β-catenin, driving pathway activation. Conversely, inhibitors such as XAV939 suppress β-catenin stabilization, while Pin1 inhibitors (Juglone and PiB) counteract Pin1-driven activation and LMP1-mediated amplification, effectively downregulating the pathway. The model highlights the therapeutic potential of targeting Pin1 in diseases characterized by hyperactive Wnt signaling, such as PF.

## 5. Discussion

Cancer has long been recognized as a major health burden worldwide, characterized by uncontrolled cell growth and the potential for metastasis (16-23). Infectious diseases, ranging from viral to bacterial infections, continue to pose significant challenges to public health, leading to substantial morbidity and mortality (24-31). In recent years, PF, a chronic and progressive lung disease characterized by the thickening and stiffening of lung tissue, has emerged as another critical health issue. The PF leads to severe respiratory impairment and has limited treatment options, highlighting the urgent need for new therapeutic strategies.

The role of Pin1 in the regulation of the Wnt/β-catenin cascade in the context of PF was studied. Our findings demonstrate that Pin1 overexpression significantly enhances the Wnt/β-catenin pathway, as evidenced by the increased levels of β-catenin, cyclin D1, and Axin2 in MRC-5 cells. Conversely, the inhibition of Pin1 with Juglone or PiB markedly suppressed this pathway, suggesting that Pin1 is a pivotal regulator of β-catenin signaling. These observations align with prior research that has identified Pin1 as a stabilizer of β-catenin, promoting its nuclear localization and transcriptional activity (3). Additionally, Pin1 inhibitors have been reported to suppress the replication of viral DNA, adding another dimension to its regulatory role in viral infection and cellular pathways (9, 32).

The Wnt/β-catenin pathway in PF has been extensively studied. Activation of this pathway is known to promote the fibrotic response by enhancing the proliferation and differentiation of fibroblasts, leading to the formation of myofibroblasts, which are key players in the deposition of the extracellular matrix (33). Our findings align with this body of research, highlighting the significant upregulation of β-catenin and its downstream targets in response to Pin1 overexpression. This suggests that Pin1 could contribute to the fibrotic process by enhancing Wnt/β-catenin signaling.

Mechanistically, Pin1 may regulate β-catenin through multiple modes. One possibility is direct isomerization at phosphorylated Ser/Thr-Pro motifs, a process known to alter β-catenin's conformation and stability. Alternatively, Pin1 may stabilize β-catenin indirectly by promoting complex formation with other cofactors that enhance its nuclear localization and transcriptional activity. Our Co-IP data further support this interaction, especially in the presence of LMP1, which significantly enhances Pin1-β-catenin binding. While enzymatic isomerization was not directly tested in this study, the observed enhancement of pathway components suggests that Pin1 functions as a molecular amplifier of β-catenin signaling.

Although the present study focused primarily on protein-level analysis, future work will incorporate RT-qPCR validation to confirm the transcriptional regulation of Wnt/β-catenin target genes and strengthen mechanistic interpretation.

Building on this, our data reveal that Pin1 not only regulates β-catenin signaling but also interacts directly with β-catenin, as confirmed by Co-IP studies. This interaction was found to be significantly enhanced in cells overexpressing the LMP1, an EBV protein implicated in various cellular processes. Furthermore, LMP1 was shown to upregulate Pin1 expression, which in turn amplified β-catenin signaling. This novel finding highlights a potential link between viral-mediated mechanisms and the progression of PF through the Wnt/β-catenin pathway. The ability of LMP1 to modulate Pin1 expression and activity further emphasizes the multifaceted role of Pin1 in cellular signaling networks.

However, it is important to emphasize that the link between EBV and PF is currently hypothetical, with no definitive clinical evidence establishing a causal relationship. While some studies have reported the presence of EBV DNA in lung tissues from patients with idiopathic pulmonary fibrosis (IPF), these findings are associative and do not prove a functional role for EBV in fibrotic disease. For instance, Tang et al. identified EBV DNA in the lungs of IPF patients, proposing a potential role in disease exacerbation (34). Padilla et al. discussed the possible association of latent viral infections, including EBV, with chronic lung diseases, suggesting an indirect contribution to fibrosis (35). Given the lack of mechanistic or longitudinal data, we present our observations involving LMP1-mediated Pin1 modulation as a preliminary in vitro model to explore this hypothesis. Further clinical studies are warranted to establish whether EBV contributes directly to PF pathogenesis.

To address concerns about the specificity of Pin1 inhibitors (Juglone and PiB), we employed several strategies. First, we performed parallel genetic approaches using siRNA-mediated Pin1 knockdown, which produced consistent results with the pharmacological inhibition, thereby reinforcing specificity. Additionally, we conducted rescue experiments wherein Pin1 overexpression was combined with inhibitor treatment. Notably, co-treatment partially reversed the inhibitory effects of Juglone and PiB on β-catenin, cyclin D1, and Axin2 levels, indicating that the observed effects are primarily mediated through Pin1 inhibition rather than non-specific redox modulation.

Furthermore, careful dose selection was performed to minimize cytotoxicity and non-specific effects, as both inhibitors are known for their potential redox activity. The consistent effects observed with both Juglone and PiB, despite their distinct mechanisms of Pin1 inhibition, support the specificity of the findings. Pin1's role as a regulator of protein function through isomerization of phosphorylated serine/threonine-proline motifs has been previously documented in various contexts, including cancer and neurodegenerative diseases (3). This study extends that understanding to PF, providing evidence that Pin1 modulation significantly alters Wnt/β-catenin signaling in lung fibroblasts.

Moreover, the discovery of EBV-LMP1's influence on Pin1 further emphasizes the potential for viral factors to exacerbate disease progression. The integration of LMP1 into this framework highlights the possibility that viral infections could act as environmental triggers or modulators of fibrotic disease processes. This is particularly relevant given the recent interest in targeting prolyl isomerases for therapeutic purposes (36).

While our findings demonstrate the potential of targeting Pin1 to modulate Wnt/β-catenin signaling in PF, several limitations should be acknowledged. The study relies on MRC-5 cells, which, although relevant, do not fully replicate the pathological environment of fibrotic lung tissue. Validation using primary fibroblasts isolated from PF patients is essential for clinical relevance. Likewise, in vivo studies — particularly in bleomycin-induced PF models — are necessary to evaluate the therapeutic potential of Pin1 inhibitors within the physiological context of the lung.

Furthermore, the study does not assess key fibrotic phenotypes, such as collagen I, α-smooth muscle actin (α-SMA), or TGF-β expression. These markers are critical for establishing a mechanistic link between Pin1-mediated β-catenin activation and fibrosis. In addition, functional assays such as proliferation, migration, and matrix deposition were not performed but will be important in future studies to substantiate the observed signaling changes.

Additionally, pharmacological inhibitors such as Juglone and PiB, though useful, may have off-target effects that could confound the results. Employing more specific genetic approaches, such as CRISPR-Cas9 mediated knockdown of Pin1, could offer more precise insights.

### 5.1. Conclusions

The current study provides compelling evidence that Pin1 plays a crucial role in regulating the Wnt/β-catenin cascade in PF. We demonstrated that Pin1 overexpression statistically augmented the Wnt/β-catenin pathway, increasing β-catenin, cyclin D1, and Axin2 in human lung fibroblast cells (MRC-5). Conversely, inhibition of Pin1 using Juglone or PiB markedly suppressed this signaling pathway, suggesting that Pin1 is a critical regulator of β-catenin signaling. Furthermore, the interaction between Pin1 and β-catenin, amplified by viral factors such as LMP1, highlights a novel mechanism linking viral influences to PF progression. While these results are based on in vitro data, Pin1 inhibitors show promising preclinical potential. However, their therapeutic relevance in PF requires further validation in primary human fibroblasts and animal models.

ijpr-24-1-160860.pdf

## Data Availability

The dataset presented in this study is available on request from the corresponding author during submission or after publication. The data are not publicly available due to ethical restrictions and institutional confidentiality policies.
